# Editorial: Radiolabeled Peptides in Cancer Imaging and Therapy—Emerging Isotopes

**DOI:** 10.3390/ph18121836

**Published:** 2025-12-02

**Authors:** Noeen Malik

**Affiliations:** CRF (Cyclotron and Radiochemistry Facility), Department of Radiology, Stanford School of Medicine, 1701 Page Mill Road, Stanford, CA 94304, USA; noeen254@stanford.edu

## 1. Overview—Precision with Purpose

Radiolabeled peptides have emerged as indispensable tools in precision oncology and molecular imaging, owing to their high target affinity, rapid tissue penetration, and favorable pharmacokinetic profiles. Their versatility continues to drive innovation across both diagnostic and therapeutic domains. This Special Issue of *Pharmaceuticals* features a curated collection of original research articles and a review showcasing the latest advances in peptide-based radiopharmaceuticals. From innovative labeling methods and dual-receptor targeting to preclinical optimization and novel applications in infectious disease imaging, these contributions represent the cutting edge of translational radiopharmacy.

Barta et al. presented a compelling in vitro method for evaluating renal uptake of radiolabeled peptides using freshly isolated rat renal cells. Their study focused on somatostatin and minigastrin analogs labeled with ^111^In and ^177^Lu, highlighting the role of megalin-mediated transport in renal accumulation. This animal study approach provides a valuable preclinical screening tool to estimate radiopharmaceutical nephrotoxicity before advancing to in vivo testing and eventual clinical use.

**Figure 1:**
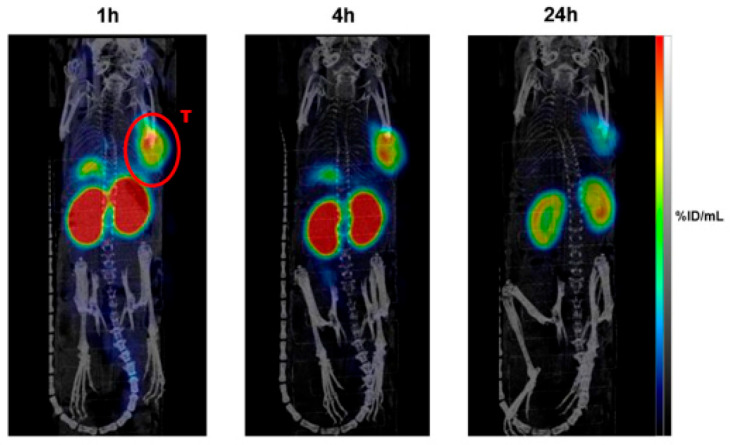
SPECT/CT scans of H69 tumor-bearing mice (*n* = 4) after administration of [^203^Pb]Pb-eSOMA-01 (Letter “T” denotes the tumor, highlighted in red and encircled).

In the context of targeted alpha therapy (TAT), Chapeau et al. introduced [^212^Pb]Pb-eSOMA-01, a radiolabeled somatostatin analog designed for neuroendocrine tumors (Figure 1). The compound demonstrated high tumor uptake and an encouraging tumor-to-kidney absorbed dose ratio in preclinical models, and its theranostic applicability was validated by co-developing the diagnostic surrogate [^203^Pb]Pb-eSOMA-01, reinforcing the potential of lead-212-based agents in advancing personalized alpha therapy for receptor-expressing tumors.

To enhance therapeutic efficacy through pharmacokinetic modulation, Szücs et al. studied NAPamide-based peptides conjugated with a 4-(p-iodophenyl)-butanoic acid (IPB) albumin-binding moiety. These compounds, radiolabeled with ^68^Ga, ^205/206^Bi, and ^177^Lu, exhibited prolonged blood circulation and improved tumor accumulation in melanoma-bearing mice. The study’s findings underline the utility of albumin-binding strategies in increasing the bioavailability and tumor residence time of peptide radiopharmaceuticals, particularly when longer-lived isotopes are employed.

Expanding the targeting repertoire, Okarvi developed and evaluated a novel angiotensin II (AngII) analog conjugated with DOTA and labeled with ^68^Ga and ^177^Lu. The compound showed high specificity for AT1-receptor-positive breast cancer cells in both ER+ and triple-negative models. Importantly, it also demonstrated fast renal clearance, making it an attractive candidate for further development as a diagnostic or therapeutic agent tailored to breast cancer subtypes overexpressing angiotensin receptors.

**Figure 2:**
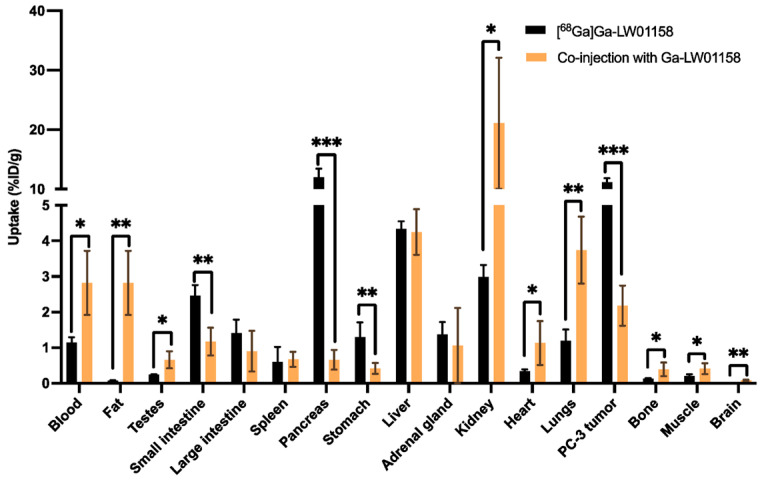
Comparison of the uptakes of [^68^Ga]Ga-LW01158 with/without co-injection of 100 µg of nonradioactive Ga-LW01158 in PC-3 tumor xenografts and major organs/tissues in mice at 1 h post-injection. Error bars indicate standard deviation (*n* = 4). * *p* < 0.05; ** *p* < 0.01; *** *p* <0.001.

Wang et al. made strides in the optimization of GRPR-targeting radioligands by engineering a suite of [^68^Ga]-labeled bombesin analogs, incorporating unnatural amino acid substitutions. These modifications enhanced in vivo stability and minimized non-specific pancreatic uptake. Among the candidates, [^68^Ga]Ga-LW01158 stood out for its superior tumor targeting and favorable biodistribution, emphasizing the potential of GRPR antagonists in prostate and breast cancer molecular imaging (Figure 2).

The comprehensive review by Leier and Wuest explored the evolution of bioconjugation chemistry in peptide radiolabeling. Moving beyond traditional copper-catalyzed click chemistry, they examined novel methods such as tyrosine-click, SuFEx, and thiol–ene reactions. These strategies offer benefits including faster kinetics, biorthogonality, and better compatibility with short-lived radionuclides, thus broadening the chemical toolkit for efficient and site-specific peptide labeling. Such innovation supports the growing need for automation and rapid tracer development in nuclear medicine.

Finally, Ferreira et al. introduced a heterobivalent peptide that combines RGD and neuropeptide Y analogs to simultaneously target αvβ3 integrins and Y1 receptors. Their dual-target radiotracer, [^99m^Tc]HYNIC-cRGDfk-NPY, showed efficient uptake in both ER+ and triple-negative breast cancer xenografts. This approach exemplifies the power of dual-receptor targeting to address tumor heterogeneity and improve diagnostic accuracy across different cancer phenotypes.

## 2. Emerging Frontiers in TAT

Peptides labeled with alpha-emitting radionuclides such as ^225^Ac and ^211^At have demonstrated promising potential in addressing therapeutic resistance and eliminating micro-metastatic disease. ^225^Ac-labeled peptides (e.g., DOTA-TOC, PSMA-617) have entered clinical studies, showing high tumor-cell lethality through double-strand DNA breaks. However, the ^225^Ac decay chain (→^221^Fr, ^217^At, etc.) involves multiple α-recoils that can break chelation bonds, causing release of daughters to off-target tissues (especially bone and kidney). Thus, challenges remain in securing stable chelation and mitigating the redistribution of daughter isotopes. On another note, ^211^At-labeled peptides benefit from being halogens, allowing direct labeling on tyrosine residues, though At-C bond stability remains formulation-dependent. Their short-range cytotoxicity, combined with a relatively short half-life and α-particle range of ~55 μm, makes them suitable for localized therapy and low-burden disease (or micrometastatic lesions).

^211^At-labeled probes have shown effective tumor targeting with minimal off-target toxicity in glioma and ovarian cancer models, demonstrated in early human glioma trials using ^211^At-ch81C6 [1]-selective uptake and tolerable toxicity using [^211^At]MX35 F(ab′)_2_ [2]; favorable pharmacokinetics and low off-target exposure in ovarian cancer models; and potent tumor suppression in preclinical FAPI-based ^211^At therapy in glioma xenografts [3]. Additionally, ^225^Ac-labeled PSMA ligands show good efficacy in treating prostate cancer. In mice, a single 40 kBq dose of ^225^Ac-PSMA-617 significantly suppressed tumor growth and extended survival [4], while ^225^Ac-RPS-074 led to complete responses in 86% of LNCaP xenografts [5]. Clinically, ^225^Ac-PSMA-617 induced >50% PSA declines in approximately 60% of mCRPC patients [6].

Since ^211^At emits few high-energy gammas (only weak emissions at ~567–897 keV and ~40% X-ray yield at ~78–90 keV), quantitative imaging is challenging and uncommon. The ^209^At/^211^At pair has been explored to enable SPECT imaging using ^209^At as a surrogate (producing the first-ever SPECT images acquired in mice) [7]. Similarly, the low photon emissions of ^225^Ac make direct imaging impractical; instead, its daughter ^221^Fr can be visualized using advanced coded-aperture or Compton imaging for preclinical biodistribution studies [8]. Surrogate-based SPECT/CT and PET strategies are increasingly validated for theranostic tracking in targeted alpha therapy.

To date, the only clinical applications of ^211^At-labeled antibodies involve intracavitary administration of ^211^At-ch81C6 in patients with recurrent glioblastoma [1] and a human intraperitoneal Phase I study with ^211^At-MX35 F(ab′)2 in ovarian cancer [2]. Another Phase I trial is underway evaluating systemic therapy with [^211^At]NaAt for refractory differentiated thyroid cancer [9]; however, no clinical trials using ^211^At-labeled antibodies or peptides for systemic administration have been reported to date. In contrast, ^225^Ac-labeled PSMA and somatostatin ligands, as well as HER2-selective pre-targeted radioimmunotherapies, are in active clinical development. Notably, ^225^Ac-PSMA-617 is being investigated in advanced Phase I/II trials for metastatic castration-resistant and hormone-sensitive prostate cancer, showing >50% PSA decline rates up to ~82% in real-world cohorts [10,11]. Additionally, ^225^Ac-DOTATATE has entered clinical evaluation in neuroendocrine neoplasms, demonstrating acceptable safety and disease control rates of ~80% [12]. A preclinical study of ^225^Ac-PRIT (pre-targeted radioimmunotherapy) targeting HER2-positive breast cancer, is also underway [13], with encouraging efficacy, supporting progression toward clinical translation. Overall, advances in peptide conjugation chemistry (as discussed by Leier and Wuest [14]) and improved isotope availability will be key to realizing the clinical potential of these agents.

## 3. Global Challenges

The clinical expansion of peptide-based radiopharmaceuticals is limited by the availability and accessibility of key diagnostic and therapeutic radionuclides. These constraints affect the production, scalability, and global distribution of nuclear medicine agents.

- **^177^Lu**: Widely used for peptide receptor radionuclide therapy (PRRT), ^177^Lu is commercially available but subject to rising demand and regional shortages.
  **Commercial Suppliers**: Eckert and Ziegler (EZAG) (nuclear-reactor-based n.c.a. ^177^Lu); ITM (EndolucinBeta^®^); SHINE (fusion-driven neutron production via Cassiopeia); Isotopia (Global cGMP facility producing n.c.a. and c.a. ^177^Lu); Bruce Power + ITM / Framatome consortium (power-reactor-based ^177^Lu production); ANSTO (OPAL-reactor-based ^177^Lu supply); NTP Radioisotopes (with ITM tech transfer) (n.c.a. ^177^Lu); MONROL (reactor-based ^177^Lu supply).
- **^68^Ga**: While widely accessible via generators, ^68^Ga is limited by its batch capacity, affecting its utility in high-throughput clinical settings.
- **^64^Cu, ^203^Pb, ^89^Zr**: These isotopes require mid- to high-energy cyclotrons, making them inaccessible in many healthcare centers without access to cyclotron or cGMP radiochemistry facilities.
- **^225^Ac**: A key radionuclide for TAT, ^225^Ac faces limited production, often relying on ^229^Th decay or high-energy proton spallation methods. Its global demand far exceeds current capacity. Several companies have announced scale-up efforts, but a large-scale stable supply remains a work in progress.
  **Commercial Suppliers**: Eckert & Ziegler (cyclotron-based irradiation onto ^226^Ra targets); NorthStar (electron-beam accelerator onto ^226^Ra targets); TerraPower (^229^Th decay harvesting in partnership with DOE); AlfaRim Medical/IONETIX Corporation (cyclotron-based ^226^Ra bombardment); PanTera Consortium (IBA and SCK CEN) (Rhodotron^®^ electron accelerator production with industrial scale); Nusano (linear-accelerator-based, upcoming); ITM (Actineer™ JV with Canadian Nuclear Laboratories) (industrial-scale production via Actineer); BWXT Medical (DMF submitted to FDA for ^225^Ac API) in agreement with NorthStar supporting ^225^Ac production; SpectronRx recently announced that it has commenced mass production of high-purity Actinium-225 (^225^Ac)
- **^211^At**: Although ideal for alpha therapy due to its short half-life and high LET, ^211^At production requires bismuth targetry and specialized cyclotrons, hindering scalability. The commercial-supplier statements below are either early-stage or under development, rather than long-established large-scale production practices.
  **Commercial Suppliers**: IONETIX Corporation (first commercial ^211^At cyclotron facility); Nusano (partnering with Atley to deliver large-scale ^211^At supply (~2025 rollout)); Framatome + IBA/PanTera (planning a European production plant (2027–2028) and to advance the industrial-scale production of ^211^At in Europe and the United States through a network of specialized cyclotrons).

These challenges are not only current but are poised to shape the radiopharmaceutical landscape over the next several years. While global demand and production of many radiometals continues to expand (Table 1), regional shortages often arise from single-source dependencies, reactor downtime, or transportation constraints rather than total global unavailability.

A comparative projection of radiometal utilization between 2025 and 2030 underscores these evolving trends. As shown in Figure 1, clinical use of ^225^Ac and ^211^At is anticipated to rise sharply due to the growing momentum of targeted α-therapy, whereas established radioisotopes such as ^177^Lu and ^68^Ga are expected to maintain steady, moderate growth as standard-of-care theranostics. Emerging PET isotopes, including ^64^Cu and ^89^Zr, are projected to gain further traction with expanding immuno-PET and theranostic applications. Collectively, these trends reinforce the urgency of addressing supply-chain bottlenecks and expanding isotope accessibility to meet escalating clinical demand.

**Figure 3:**
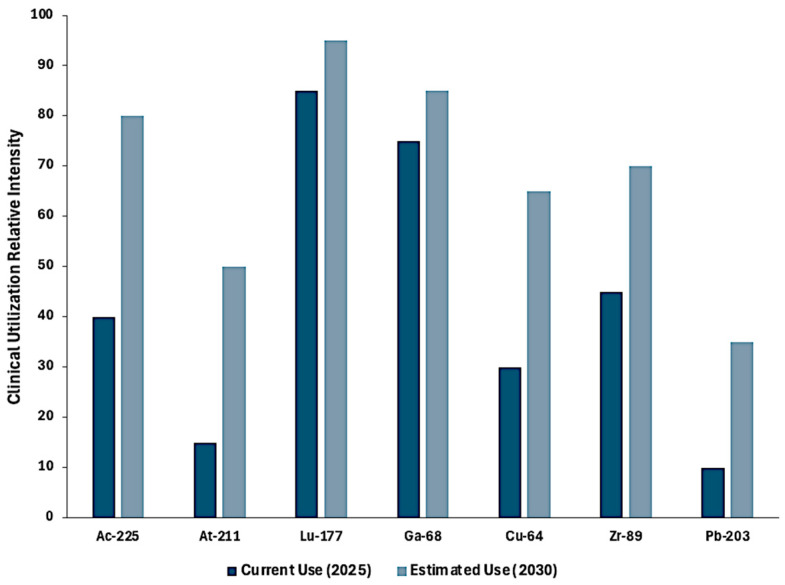
Projected growth of radiometals in clinical applications (2025–2030).

The clinical utilization projections in Figure 3 are based on a structured expert estimation model integrating qualitative and semi-quantitative indicators. Radiometals were scored on a 0–100 scale reflecting anticipated utilization intensity between 2025 and 2030, considering regulatory progress (trial phase and approvals), production availability (infrastructure and supply), theranostic versatility (application breadth and imaging compatibility), scientific momentum (literature presence and strategic prioritization), and market forecasts. These variables were synthesized from IAEA reports, peer-reviewed reviews [15,16,17,18,19,20], and industry analyses, and final scores were refined through an informal Delphi-style consensus process based on production feasibility, clinical momentum, and translational maturity as of 2024.

Recent sources estimate the global alpha-emitters market to have a values of around USD 678 million in 2023, growing at a projected compound annual growth rate of ~11–20% CAGR to reach USD 1.4–3.2 billion by 2030 [21]. Companies such as POINT Biopharma, Telix Pharmaceuticals, IONETIX, and SHINE are investing in production capacity and clinical deployment pipelines. ^211^At continues to receive scientific interest due to its favorable decay properties and promising clinical applications; however, its broader clinical adoption remains constrained by limited production infrastructure and the need for high-energy cyclotrons for bismuth target irradiation [22]. Regulatory incentives, such as orphan drug status and government funding, are accelerating adoption in both the U.S. and Europe.

These availability constraints necessitate the following measures:

- Investment in alternative production routes (e.g., cyclotron-based ^225^Ac, thorium target irradiation).
- Expansion of generator-based platforms, particularly for ^68^Ga and ^203^Pb.
- International collaboration and harmonization of isotope distribution and regulatory approvals.
- Development of flexible radiochemistry platforms to adapt tracers for multiple isotopes based on local availability.

To enable equitable global use of peptide radiopharmaceuticals, it is essential to develop alternative production routes, invest in generator-based supply chains, and expand international isotope infrastructure.

## 4. Coda

The advancements highlighted in this Special Issue reflect a defining moment in the trajectory of radiolabeled peptide development. As the field transitions from innovation to integration, the convergence of molecular design, radiochemistry, and translational research is unlocking new opportunities for targeted imaging and therapy. The maturation of alpha-emitting agents, dual-receptor tracers, and novel bioconjugation strategies offers compelling evidence of progress toward increasingly precise and personalized interventions. Yet these scientific gains must be matched by coordinated efforts to overcome persistent barriers in isotope production and global distribution. Bridging these gaps will be essential to ensure that the clinical potential of radiolabeled peptides is fully realized—not as isolated breakthroughs, but as standardized tools across healthcare systems. The future of molecular radiotherapy depends not only on continued innovation, but on scalable, equitable implementation that delivers on the promise of precision medicine.

## Figures and Tables

**Table 1:** Forecasted market growth and clinical readiness of radiometals (2025–2035) [14,15,16,17,18,19].

Radionuclide	Market Outlook/Forecast	Clinical Activity Level
^177^Lu	Market projected to grow from USD 2.73 billion (2025) to USD 10.84 billion (2032) (~21.8% CAGR)	Highest; FDA-approved for NETs (Lutathera) and prostate cancer (Pluvicto)
^225^Ac	Market forecast to grow from ~USD 0.7 million (2023) to USD 1.7 billion (2031) per verified market research (2023); numerous Phase I/II trials	High and expanding rapidly
^68^Ga	Rising PET theranostic use (e.g., PSMA-11, DOTATATE); integral to expanding radiotheranostics market, projected to reach USD 31.8 billion by 2035	High; widely used in PET imaging
^89^Zr	Niche but expanding for immuno-PET; several active antibody-tracking trials	Moderate; used in clinical trials
^64^Cu	Used in diagnostics (^64^Cu-DOTATATE, ^64^Cu-PSMA) and as theranostic pair with ^67^Cu; several trials	Moderate; expanding theranostic interest
^203^Pb	Diagnostic surrogate for ^212^Pb; early clinical evaluations as imaging companion in TAT development	Low to moderate; companion imaging use
^211^At	Early-stage α-emitter under evaluation in first-in-human glioblastoma and thyroid cancer trials; not yet in routine clinical use	Low; not yet in routine clinical use
